# Towards Safer Electric Vehicles: Autoencoder-Based Fault Detection Method for High-Voltage Lithium-Ion Battery Packs

**DOI:** 10.3390/s25051369

**Published:** 2025-02-23

**Authors:** Grzegorz Wójcik, Piotr Przystałka

**Affiliations:** 1 Department of Fundamentals of Machinery Design, Silesian University of Technology, 18a Konarskiego Street, 44-100 Gliwice, Poland; grzegorz.wojcik@draexlmaier.com; 2 DIP Draexlmaier Engineering Polska Sp. z o.o., 44-100 Gliwice, Poland

**Keywords:** electric vehicles, battery packs, liquid detection, machine learning

## Abstract

The rapid growth in the battery electric vehicle (BEV) market has brought lithium-ion battery (LIB) packs to the forefront due to their superior power and energy density properties. However, LIBs are highly susceptible to environmental factors, operating conditions, and manufacturing inconsistencies and operate within a narrow safety operating window. Battery faults pose significant risks, including potentially catastrophic thermal runaway, that can be initiated even by small faults, propagating further into a chain reaction cascade of failures. Aiming to improve the safety of such battery packs, this article presents the developed autoencoder-based fault detection method. The method, enhanced by computational intelligence and machine learning, is a result of extensive research into optical liquid detection systems (OLDSs) for immersion-cooled battery packs, where optical rather than electrical signals are used inside high-voltage areas. The performance was evaluated using recorded real-life datasets under faultless states and under simulated fault states through specific model performance indicators as well as detection performance indicators.

## 1. Introduction

In the past fifteen years, the electro-mobility market has seen a significant surge. Between 2013 and 2018, the worldwide count of electric passenger cars escalated from 0.3 million to over 5 million, witnessing a remarkable rise of 2.1 million in 2018 alone. This figure has multiplied eightfold in a short span of four years, reaching over 40 million by the close of 2023. In 2023, battery electric vehicles (BEVs) accounted for over two-thirds of all electric car sales globally, and it is projected that lithium-ion batteries (LIBs) will continue to be the predominant choice in the electric vehicle (EV) market for the forthcoming decade [1,2,3]. Despite the advancements in LIBs that enhance their longevity, factors such as environmental conditions and usage patterns are key determinants of their lifespan. LIBs are particularly susceptible to reduced lifespan due to high rates of charging and discharging, especially under extreme ambient temperatures [4]. Operating these batteries within a specific temperature range, from −20 °C to +60 °C, is crucial to prevent damage, and optimal performance is observed when the cell temperature is maintained around +20 °C [5]. As a result, modern battery packs incorporate both battery management systems (BMSs) and battery thermal management (BTM) systems. These systems help avert rapid aging or damage to the battery cells through various means such as cell balancing, charging regulation, and thermal management, along with monitoring the state of charge (SOC) and state of health (SOH) [6]. With the diverse range of ambient temperatures encountered around the world, EV battery packs are designed to endure both low and high operational temperatures. Car manufacturers achieve superior performance by implementing complex cooling mechanisms and architectures optimized for efficiency, reducing power losses in the system. Moreover, in many BEVs, the battery pack also functions as part of the vehicle’s chassis, necessitating a design that ensures mechanical stability over the EV’s lifetime. This includes the use of specialized sealants, adhesives, and thermal interface materials.

The shift towards car electrification has catalyzed the emergence of novel technologies as well as the adaptation of existing ones to meet new requirements. Replacing internal combustion engines with electric motors and high-density battery packs has brought about a host of new opportunities but also introduced a range of challenges to address. Among the most critical of these challenges are thermal runaway and thermal propagation events, as reported in several studies [6,7,8]. The escalating demand for lithium-ion battery packs has been paralleled by rare, yet highly publicized, fire incidents. These incidents have not only gained attention beyond local regions but have also necessitated widespread recall and inspection initiatives. These measures are often taken proactively or in compliance with government directives, resulting in financial and reputational damage for the manufacturers involved. Recent research has proposed various strategies to mitigate the risks associated with these fire incidents. These include enhancing the thermal stability of the battery cells, reducing the heat transfer between cells, and implementing early detection systems for battery faults. These detection systems are particularly important as they can provide early warnings and allow for necessary evacuation measures, thereby reducing potential harm [9].

The severity of thermal propagation events in electric vehicles has prompted the Global Technical Regulations (GTRs) to propose a comprehensive regulatory framework that includes a detailed thermal propagation test procedure. This procedure, designed to enhance occupant safety, encompasses various levels including the cell, module, pack, and the vehicle itself, especially in the event of thermal runaway within the battery pack system [10]. The test simulates thermal propagation initiated by a thermal runaway in a single cell, caused by an internal short circuit. The GTR mandates that a warning system must alert the vehicle’s occupants at least five minutes before the onset of a thermal propagation event.

Typically, BEVs employ 400 V battery packs, but recent technological advancements have seen the introduction of 800 V systems [11]. When these LIBs undergo rapid charging or are subject to quick acceleration, they can endure charge and discharge cycles involving hundreds of amperes. However, due to the suboptimal characteristics of system components (e.g., module and cell connections), such high currents can lead to considerable heat accumulation within the cells, negatively impacting the battery pack’s performance. In response to the growing demand for improved performance, novel advancements in BTM are being explored. One such innovation is the development of fully immersed LIB packs, offering superior thermal dissipation capabilities compared to traditional air cooling methods. Moreover, these systems are adept at equalizing temperature variations across the battery pack, thereby enhancing overall efficiency and safety [12]. Although complex in both, design and implementation, such advanced battery thermal management systems have been successfully implemented in several high-profile vehicles. This is exemplified in models such as the McLaren Speedtail, Mercedes AMG GT 63 S E Performance, and the Faraday Future FF91 [11,13,14].

The introduction of battery packs equipped with immersion cooling technology represents a cutting-edge development in BTM, yet this novelty also means that not all potential complications have been addressed in advance. Despite extensive research efforts focused on understanding and mitigating a variety of faults in EV battery systems, a notable gap remains in the study of liquid leakage and intrusion faults. These faults, typically involving dielectric fluids like mineral oil and water intrusion, are critically important due to their potential to significantly impair battery performance [15] and compromise safety [16,17]. Surprisingly, they have not garnered as much academic attention as other types of faults. Leakage of such fluids can undermine the efficiency of the BTM system, while water intrusion can lead to short circuits and corrosion.

As the design of battery packs evolves, the integration of liquid-sensing devices has become more common to enhance safety and performance measures. These sensors typically function by detecting changes in electrical resistance when their electrodes encounter a conductive liquid. While they are proficient at identifying the presence of conductive fluids, a limitation is their inability to distinguish between different types of liquids; they simply trigger an alert upon any detection. Moreover, these sensors may require replacement after detecting water intrusion due to electrode corrosion, which could increase the overall maintenance demands of the system [18]. From a practical standpoint, incorporating these sensors into a battery pack system necessitates thoughtful planning. Drawing from the authors’ experience, it is essential to ensure galvanic isolation or adequate protection of the power supply and communication lines that connect the BMS, operating at a low voltage, with the high-voltage battery.

Research indicates that intensity-based polymer optical fiber (POF) sensors show promise in detecting liquid intrusion and identifying the specific type of fluid, whether air, water, or oil [19,20,21,22,23,24,25]. This characteristic makes POF sensors particularly suitable for fully immersed battery systems, where they can simultaneously monitor both liquid leakage and intrusion. An added advantage of these sensors is their lack of direct electronics–liquid contact, which enhances system reliability compared to electrical sensor alternatives. However, the relatively rare application of polymer optical fiber sensors in the automotive industry raises concerns about their durability and efficacy under typical vehicular conditions. Variables such as extreme temperature ranges (from −40 °C to +85 °C), constant vibrations, and the need for a prolonged operational life (spanning 10–20 years) represent potential challenges for their deployment in vehicle systems. Moreover, developing sophisticated methods for processing and interpreting data from POF sensors is necessary to effectively identify symptoms of the aforementioned faults [26]. Addressing these concerns is important to ensure the practical viability of optical fiber sensors in automotive battery thermal management systems.

The increasing need for high-performance battery packs has led car manufacturers to boost the charging and discharging currents to unprecedented levels. As a result, battery thermal management systems are continually evolving to maintain safe operational conditions and minimize aging effects. Despite these advances, battery packs remain vulnerable to a range of faults, from system-wide issues like sensor, cell connection, and cooling system failures to individual cell problems such as overcharging, overdischarging, and short-circuits, which can potentially trigger a cascaded reaction, ultimately leading to fire and explosion [27]. While extensive research has been directed towards understanding and addressing these faults, the specific areas of liquid leakage and intrusion remain notably under-researched, despite their significant impact on battery performance and safety. This oversight represents a research gap that needs addressing. Current findings suggest a pressing need for advanced prevention mechanisms to enhance safety and provide timely warnings of hazardous conditions within battery packs. These mechanisms should be designed to function reliably in high-voltage environments prone to electromagnetic interference and electrostatic discharges, potentially utilizing optical rather than electrical signals to ensure safer system monitoring in automotive conditions.

The following paper proposes a fault detection method that was designed for, but is not limited to, liquid leakage and liquid intrusion detection inside high-voltage, immersion-cooled LIB packs. This method emerges from thorough research into OLDSs, aiming to increase the safety of the aforementioned battery packs without exposing low-voltage sensing devices to hazardous, high-voltage environments within the battery pack system. The approach involves a mechatronic platform leveraging optical sensors, particularly evanescent-wave absorption polymer optical fiber sensors, distributed within the high-voltage areas of the battery pack, which are immersed in dielectric mineral oil. The research process encompassed extensive testing over a prolonged period, namely, from December 2022 to May 2023. This duration allowed for comprehensive data collection, important for the subsequent implementation and validation of the fault detection method.

The rest of the paper is organized as follows: Section 2, ‘Related Work’, outlines the existing research on fault diagnosis within electric vehicle battery packs, with a focus on the use of machine learning techniques in the automotive industry. Section 3, ‘Materials and Methods’, is structured into three parts. The first part discusses the concept of the proposed OLDS and provides an overview of the POF sensor integral to the OLDS. The second part details the system’s implementation under real-world conditions, the experiments conducted, and the data acquisition process for the research on the fault detection method. The third part describes the fault detection method developed, including the metrics used for its evaluation. Section 4, ‘Experiments and Results’, is divided into two sections; the first details the structure of the artificial neural network used, and the second presents the overall results of the fault detection algorithm. Section 5, ‘Discussion’, analyzes and interprets the results in depth. Finally, Section 6, ‘Conclusions’, summarizes the findings and implications of the optical liquid detection system for high-voltage battery packs.

## 2. Related Work

Battery faults can lead to serious hazards, but the risks associated with LIB packs can be mitigated through the safety functions of the BMS. The BMS controls critical parameters like voltage, current, and temperature, informed by sensor data, to minimize the likelihood and severity of faults. Given the complexity and variable degradation mechanisms of lithium-ion batteries, extracting fault symptoms is challenging due to cell inconsistency and hysteresis, often making standard safety measures insufficient. These measures are often not sufficient and fault diagnostic algorithms are required for the BMS. Such algorithms are often divided into two subcategories: model-based (including state estimation, parameter estimation, parity space, and structural analysis) and non-model-based (including signal processing- and knowledge-based methods) [6].

Model-based approaches use relationships among measured variables, modeled through equations or rules, to identify changes indicative of faults. They generate analytical symptoms by comparing special features such as parameters, state variables, and residuals against nominal values, thus facilitating fault detection. In automotive applications, these methods leverage battery pack models to produce residuals and are favored for their simplicity and cost-effectiveness. Non-model-based methods rely on signal processing or knowledge-based techniques and do not require intricate battery modeling. These methods, particularly data-driven ones, can predict battery behavior over time and support model development. While signal processing algorithms are dynamically robust, they struggle with measurement noise and early fault detection. Knowledge-based methods, including expert systems, fuzzy logic, and neural networks, use accumulated data or expert observations to establish rules or train models for precise fault detection. However, these systems demand extensive system knowledge, which can be challenging given the incomplete understanding of all battery faults.

More complex knowledge-based methods, such as machine learning (ML), streamline fault diagnosis by bypassing the need to gather physical battery details and learning the complex relationships between internal and external battery parameters. Although ML techniques offer high accuracy and handle the nonlinearity of lithium-ion batteries well, they require extensive data and a lengthy training process [6].

A review performed by Samanta et al. [28] provides an overview of ML-based techniques, that have been successfully used in LIB BMSs. The overview lists multiple examples, where the BMS employs one of the following:

- A combination of recurrent neural network (RNN) and long short-term memory (LSTM) structures for voltage prediction and fault prognosis;
- A support vector machine to diagnose the battery pack connection fault’s in series-connected lithium-ion cells;
- The logistic regression technique for overcharge and overdischarge fault diagnosis in nickel–metal hydride battery packs;
- Gaussian process regression for LIB state-of-health estimation for high-accuracy and degradation mode diagnosis;
- A combination of LSTM and an autoencoder for state-of-charge estimation, which could be further used for the evaluation of overcharge, overdischarge, and accelerated degradation faults.

Research on battery pack fault diagnosis [27,28,29,30,31,32,33,34,35,36,37,38,39,40,41] has revealed that the main areas of interest focus on thermal behavior, sensor performance, and assessments of battery degradation and parameters like state of charge and state of health. However, there seems to be a lack of studies specifically targeting the performance or fault detection capabilities of cooling systems, indicating another research gap. Additionally, studies have demonstrated the effective use of various machine learning techniques for parameter estimation and fault detection without the need for complex system modeling. This is particularly important for modern LIB packs that utilize direct liquid-cooling systems, which are not yet widely implemented, and therefore, their design and operational frameworks are not well understood. Given these factors, employing a machine learning approach for the developed optical liquid detection system, which uses real-world data from various operational scenarios, would be a practical strategy to enhance diagnostic capabilities and system understanding.

## 3. Materials and Methods

### 3.1. Optical Liquid Detection System

**Figure 1:**
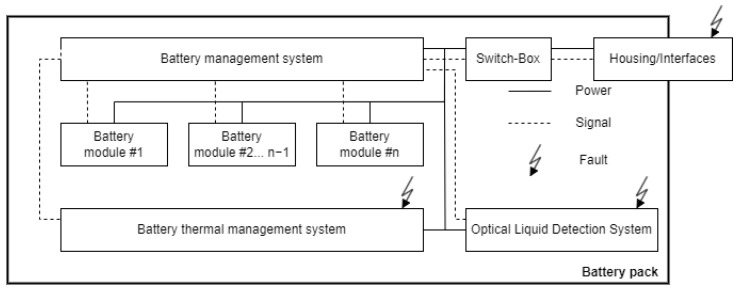
Simplified block diagram of the electric vehicle’s battery pack, with optical liquid detection system and faults.

A block diagram of the EV’s battery pack, incorporating the OLDS, is shown in Figure 1. Although every component in the diagram is susceptible to various faults, the OLDS specifically targets the detection of two particular faults: liquid intrusion and liquid leakage. These faults are highlighted in red in the diagram.

**Figure 2:**
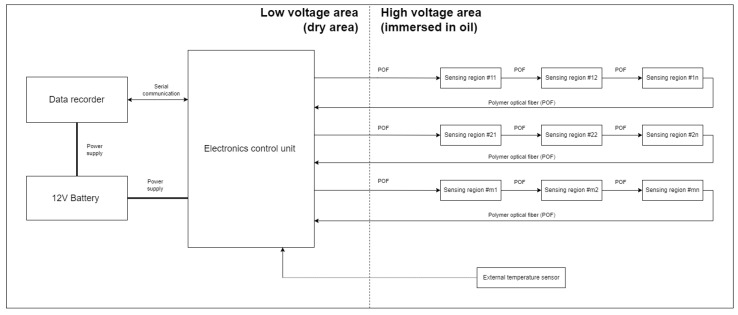
Block diagram of a simplified battery module utilizing an optical liquid detection system.

Due to the considerable size, weight, and cost of electric vehicle battery packs, the battery pack used for this research was intentionally simplified. During the tests, a housing of a single battery module, equipped with the developed OLDS, was used. For the housing, a real housing of a series-production electric vehicle was used. The setup, as shown in Figure 2, includes the battery unit in the low-voltage area along with a data recorder and an electronic control unit (ECU). The developed ECU can have up to four POF sensors connected and one external temperature sensor. The POF cables are guided from the low-voltage area to the high-voltage area, where sensing regions are immersed in the coolant (oil) to monitor the liquid’s condition. The external temperature sensor (thermistor) is situated in the high-voltage area to provide additional data about the oil temperature. These data, however, were not meant to be used by the fault detection algorithms developed in this study. They were purely for representative and debugging purposes. During the tests, only 1 POF sensor with one sensing area was used. The high- and low-voltage areas are named in a such way only for representative purposes—due to safety reasons, during the development and tests no high voltage was used and the system was powered by a 12 V car battery.

The primary objective of the optical liquid detection system was to identify faults, specifically oil leakage or water intrusion. Those technical states are listed in Table 1.

The table lists three distinct technical states of the battery pack system, categorized as faultless (F0) and two fault states (F1 and F2), within the framework of this simplified battery pack system.

- Faultless state (F0): This state reflects the battery pack system operating within its specified functionality and meeting all requirements, with no deviations from expected performance.
- Oil leakage state (F1): This state is characterized by unauthorized deviations in coolant levels, potentially due to damage to the battery pack’s structure or sealings. Initially, such a fault could lead to irregularities in the performance of the battery’s thermal management system.
- Water intrusion state (F2): In this state, water has entered the system, possibly through a compromised heat exchanger or as a result of condensation. Initially, this could lead to corrosion, create short-circuits, or damage other components through the formation of ice particles in low operating temperatures.

**Figure 3:**
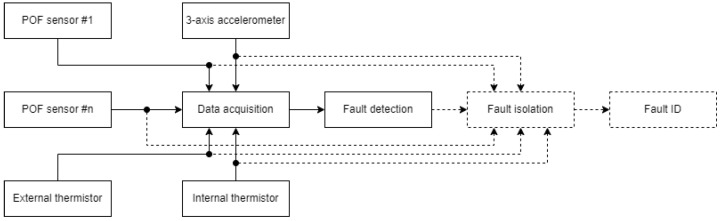
Block diagram of the data processing.

The data processing strategy for detecting relevant faults is outlined in Figure 3. This strategy involves collecting data from various sensors, including a POF sensor, a 3-axis accelerometer, and both internal and external thermistors.

The system evaluates the gathered data to monitor for any unauthorized deviations from normal operations. If such deviations are detected, the system identifies the presence of a fault and determines the time period during which it occurred. The figure further illustrates, through blocks outlined with dashed lines, that the system is designed to potentially determine the type, location, and time of detection of a fault, as well as assign a fault ID. However, these functionalities are not covered within the scope of this research. Furthermore, while not every sensor mentioned is essential for executing a robust fault detection algorithm, the inclusion of their data aids in the understanding of the system’s behavior and its responses to various fault conditions.

The OLDS developed for this study was designed with a set of specific functional and non-functional requirements tailored to enhance detecting liquid-related faults within automotive battery packs.

### 3.2. Functional Requirements

The system’s functional requirements are as follows:

- Be able to continuously monitor its operational state through measurements obtained from polymer optical fiber sensors, capable of detecting faultless, water intrusion, and oil leakage conditions.
- Be able to control the forward current for up to four POF transmitters (LEDs).
- Be able to measure the output signals from up to four POF receivers, which could be either photodiodes or phototransistors.
- Be able to monitor the temperature within the high-voltage area using external temperature measurement tools.
- Be able to monitor vibrations at the electronic control unit (ECU) level to ensure stability and accuracy in readings.

### 3.3. Non-Functional Requirements

The system’s non-functional requirements are as follows:

- Achieve a fault detection time no longer than 300 s.
- Present a true detection rate of at least 80% to ensure reliability.
- Present a false detection rate not higher than 2%, minimizing unnecessary responses and maintenance.
- Remain fully operational within a 9 V to 16 V input voltage range, accommodating a standard vehicle power system.
- Be able to function within an operating temperature range of −40 °C to +85 °C, accounting for extreme environmental conditions and self-heating.
- Be able to distinguish between fluid types across a range of refractive indices from 1.00 nD to 1.47 nD, enhancing its diagnostic capabilities.
- Reach a technology readiness level of 5, indicating a moderate level of technology maturity.
- Feature compatibility with serial-communication interfaces such as the LIN bus.

### 3.4. Data Description

**Figure 4:**
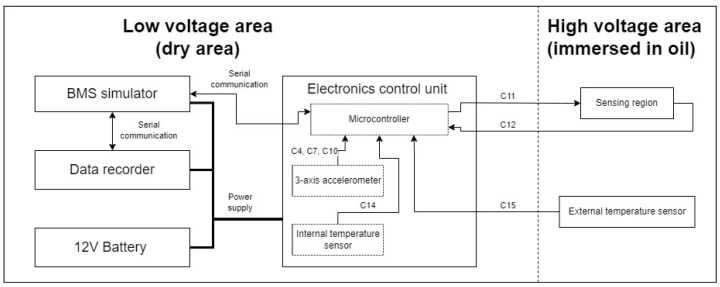
Block diagram of a simplified battery module utilizing an optical liquid detection system during in-field tests.

For typical BEVs, their battery packs are usually heavy, sizable, and expensive devices. Because of that, significant simplifications were introduced to the mechatronic platform that was used throughout the development and validation phases of this research (Figure 4). The platform utilized a real housing of a single battery module. Usually, such a battery module is a low-voltage device, and the high-voltage area is generated at the battery pack level (thanks to multiple modules connected in series). For the sake of this research, the areas within the single battery module were designated as low-voltage and high-voltage areas. However, it is important to note that, for safety reasons, no high voltage was applied during the development or testing phases. Instead, the system was powered by a 12 V battery. Operating at high voltages with a large number of cells introduces significant risks, particularly when conducting tests that involve water intrusion into the battery pack module. The presence of water raises the potential for corrosion and short circuits, which could lead to hazardous situations, including thermal runaway and thermal propagation events. Moreover, for the objectives of this initial field testing, the application of high voltage was not necessary. The primary aim of the tests was to assess the performance of the OLDS, particularly its ability to detect oil leakage and water intrusion using a polymer optical fiber refractive index sensor. This type of sensor operates based on optical signals, which are immune to electrical noise and interference, thus eliminating the need for high voltage in the evaluation of sensor performance.

**Figure 5:**
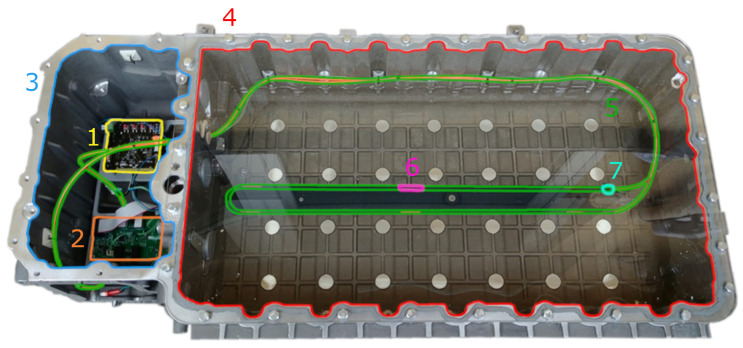
Optical liquid detection system used during the tests under road conditions.

The optical liquid detection system was assembled in the lower section of a real battery pack housing, as presented in Figure 5. This was conducted in strict accordance with the proposed system architecture, illustrated in Figure 4. Two key elements of the system, namely, the data recording device (1) and the electronic control unit (2), were placed within a dry, low-voltage area (3) of the system. The high-voltage region (4) was designed for complete immersion in oil, serving as the designated zone for fault injection experiments. This zone was occupied with a fabricated polymer optical fiber sensor (5) and an external thermistor (7), responsible for monitoring the temperature within the immersed environment.

The routing of the POF cable and external thermistor from the high- to low-voltage areas was carefully managed to maintain the integrity of the system. Given the original battery pack housing was not equipped with the necessary provisions for liquid-sensing devices, additional mounting components were necessary. These were designed, 3D-printed using PET-G material, and subsequently incorporated into the system. These components play a critical role in preventing undesired movements of the POF cables that could potentially affect the sensor’s performance. The housings incorporate small openings, strategically engineered to expose the polymer optical fiber cable and its sensing area (6) to the external medium, in this case, oil. As for the system’s power requirements, a 12 V automotive battery was utilized. Given the spatial constraints within the battery pack housing, the power supply unit was located externally. In future work, a BMS simulator will be used to execute fault diagnostic methods based on data provided by the ECU and to transmit this information to the data recorder. During the research phase, however, the BMS simulator was not in operation, and serial communication was directly linked between the ECU and the data recorder. To accelerate the research, the fault detection methods themselves were implemented and evaluated offline, using registered datasets.

**Figure 6:**
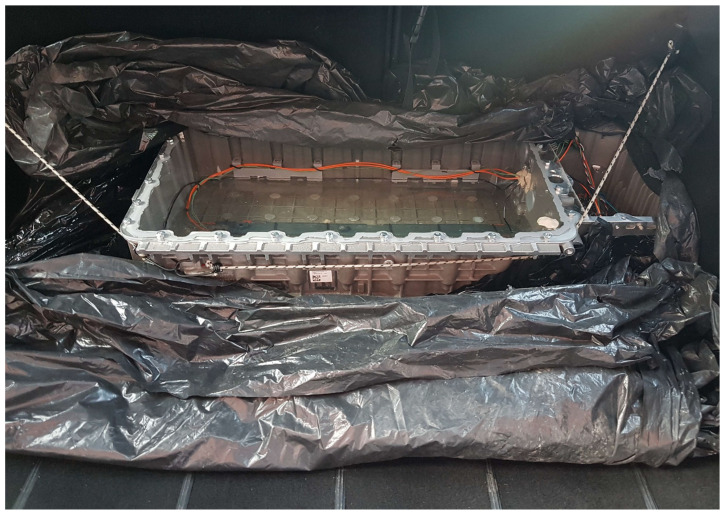
Optical liquid detection system installed inside the vehicle.

To maintain stability and minimize the risk of skewed data due to unintended movements, the system was carefully installed in the trunk of a vehicle for the duration of the tests (Figure 6). This setup was essential to ensure the integrity of the data collected under various real-world conditions. To maintain the stability of the OLDS, flexible ropes were employed to secure it. This setup ensured that the system stayed firmly in position during the operation of the vehicle, preventing any displacement caused by the vehicle’s movement. The vehicle was used in a typical daily routine throughout the testing phase, which included commuting to and from an office, along with travel on both highways and local roads. This strategy was chosen to replicate the everyday experiences of a standard vehicle, without implementing any specific or unusual driving patterns. The testing was conducted in the Silesian region of Poland, an area characterized by a variety of road types. This geographical consistency in testing was beneficial in enriching the dataset. The real-world scenarios encountered during on-road testing were diverse, involving challenges such as a wide range of operating temperatures and vibrations, typical of standard vehicle operation.

Additionally, the extended duration of the tests, spanning several months, introduced additional complexity to the research. Factors like component aging and a broad range of environmental temperatures had a significant impact on the measurements. These effects made data interpretation and processing more challenging, as they added layers of variability that had to be accurately accounted for in the fault detection system. The practical implications of these real-world testing conditions were crucial in validating the robustness and effectiveness of the proposed diagnostic methodology. Because of different thermal inertia for low- and high-voltage areas, the mechatronic platform incorporated both internal and external thermistors, offering thorough temperature monitoring capabilities. The internal thermistor was placed inside the ECU, to consistently record its temperature. In contrast, the external thermistor was immersed in the oil, near the polymer optical fiber’s sensing area. The POF sensor itself was excited using an LED transmitter, driven with constant current, and was configured to feed its measurement data back to the ECU. Considering that the heat capacity is intrinsically linked to the total volume of the liquid used, this configuration provided a comprehensive insight into the operational conditions surrounding the POF sensor.

To gather extensive and representative data, the collection periods for each dataset varied, ranging from several hours to as long as sixty hours. This approach was strategic, intended to capture the potential variations in daily temperature and assess how these thermal fluctuations might impact the sensor measurements. Additionally, to accurately record the vibrations that the OLDS was subjected to during these tests, a 3-axis accelerometer was employed, providing detailed acceleration measurements along the x, y, and z-axes. This comprehensive approach, combining temperature and vibration data, was important to understand and analyze the behavior of the system and to develop a fault detection system capable of performing reliably under real-world vehicular conditions.

From a process diagnostic point of view, there were several process variables (channels) to handle internal and external devices:

- C04(*k*), C07(*k*), C10(*k*)—accelerations measured along the x, y, and z-axes, respectively (using a MEMS accelerometer with ±8 g range, sensitivity scale factor of $8192\frac{\mathrm{LSB}}{g}$, and temperature sensitivity of ±$0.026\frac{\%}{{}^{\circ}C}$);
- C11(*k*)—POF sensor’s input signal;
- C12(*k*)—POF sensor’s output signal;
- C14(*k*)—internal thermistor’s resistance;
- C15(*k*)—external thermistor’s resistance.

The testing methodology employed for the mechatronic platform was designed to mimic real-world conditions as closely as possible. While the vehicle remained primarily stationary throughout the day, it experienced intermittent periods of motion. These movements typically lasted for several dozen minutes and were essential in providing a realistic testing environment. Such periods of vehicle movement ensured that the OLDS was also tested under dynamic conditions. This strategy of data collection allowed the system to be exposed to a spectrum of conditions, including both stationary and moving conditions, offering a comprehensive understanding of the system’s performance under three different technical states:

- Faultless state (F0)—with the high-voltage area filled with 16500 mL of dielectric liquid (mineral oil).
- Oil leakage fault (F1)—after finishing the F0 experiment, this fault was simulated by leaking oil of a volume $Q_{1}$.
- Water intrusion fault (F2)—after the completion of the F1 experiment, the water intrusion fault was injected (with variable fault magnitudes). Once intruded, the intruded water remained during further experiment scenarios and accumulated with each consecutive water intrusion occurrence.

The research encompassed 21 experiments in total that were categorized based on the technical state of the mechatronic platform. Under faultless conditions (F0), 7 experiments were conducted. Under simulated faults, 5 experiments were carried out that involved an oil leakage fault (F1), and 9 experiments were carried out that involved a water intrusion fault (F2). A parametric summary of the registered data is presented in Table 2. In this setup, the $Q_{1}$ parameter was used to quantify the magnitude of the simulated faults, measured in milliliters. For the oil leakage fault experiments, the magnitude of the fault (i.e., the amount of leaked oil) was constant. For the water intrusion fault experiments, however, the $Q_{1}$ value varied, simulating different degrees of intrusion severity. The duration of each experiment, denoted as $t_{1}$, is recorded in minutes, providing insight into the length of each test scenario. Additionally, the $t_{2}$ value represents the total duration for which the vehicle was in motion during each experiment. This motion duration was determined through post-processing of the data, based on an analysis of the logged accelerometer readings. One notable aspect is that one particular experiment under faultless conditions (F0, experiment no. 6) was conducted with the vehicle remaining entirely stationary. This lack of movement provided a unique overview, allowing for the evaluation of the system’s performance in an environment subjected mostly to temperature variations.

For the F1 experiments, the volume of leaked oil $Q_{1}$ was set at 15,000 mL. This volume was chosen to replicate a scenario where the sensor, under normal, non-moving conditions, remains fully submerged in oil. However, the dynamics change once the vehicle begins to move. Particularly during turns, the oil’s distribution inside the container is altered due to centrifugal forces, potentially exposing the sensing region to air. The substantial volume of oil was necessary because only one POF sensor was used by the mechatronic platform, and it was placed at the very bottom of the battery housing. With a single-sensor setup, a choice had to be made between prioritizing early-stage water intrusion detection or optimizing oil leakage detection. Given the experimental nature of this study, it was decided to focus on understanding the system’s fundamental capabilities with the simplest setup before expanding to multi-sensor configurations in future research. This placement results in a trade-off for oil leakage detection. A relatively large amount of oil must be leaked before the system can identify the fault, as the sensor remains submerged until the oil level drops significantly. Regarding the F2 experiments, varying fault magnitudes were introduced to assess the system’s reaction to different levels of severity. The initial fault magnitude was set at a minimal volume of 50 mL, equating to an approximate weight-to-weight concentration of 0.3%. Subsequent increases in the water intrusion magnitude were introduced in a step-wise fashion. The first increase brought the fault magnitude to 150 mL, followed by another increase, reaching the final fault magnitude of 300 mL. This gradual escalation in the fault magnitude enabled a systematic exploration of the system’s detection capabilities and its responsiveness to progressively severe conditions of water intrusion.

The comprehensive data acquisition process resulted in a substantial amount of data, amounting to a total of approximately 140,532,540 data entries. Such an amount of data entries corresponds to approximately 390 h of total experiment duration, offering a rich source of information for analysis and model training. To effectively utilize this extensive dataset, it was categorized into three distinct sets: training data (exp. IDs 5 and 6), testing data (exp.ID no. 6), and verification data (remaining experiments).

**Figure 7:**
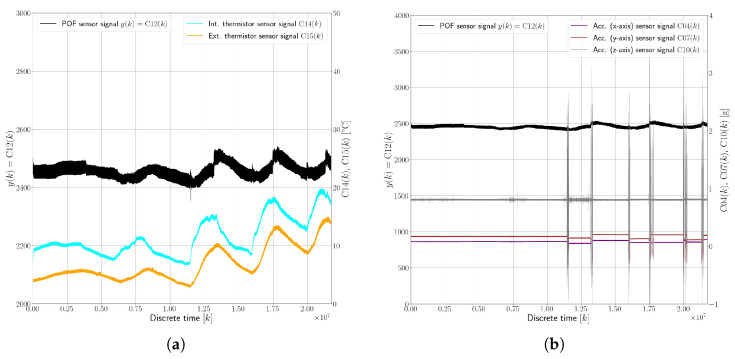
Example measurements y(k) for experiment no. 4 (F0 state) versus (**a**) temperature and (**b**) accelerations.

Figure 7 presents an example dataset, visualizing the behavior of the evanescent-wave absorption polymer optical fiber sensor and its interactions with various environmental factors under faultless conditions (F0).

This dataset, spanning 3623 min (approximately 60 h), offers insights into the sensor’s response to both ambient temperature changes and vehicle movement. Figure 7a illustrates the relationship between the sensor’s response, denoted as C12(k), and the oil temperature, indicated by C15(k). The chart shows how the sensor’s performance can vary with changes in the temperature of the surrounding medium (in this case, oil). Figure 7b displays the sensor’s response versus accelerations experienced by the vehicle, represented by C04(k), C07(k), and C10(k). These measurements are important for understanding how vehicular movements and vibrations influence the sensor’s readings. Key observations taken from the aforementioned figures include the following:

- The internal thermistor C14(k) in the ECU recorded temperatures ranging from a minimum of 5 °C to a maximum of 20 °C.
- The external thermistor C15(k), immersed in oil, registered temperatures between 3 °C and 15 °C.
- A temperature fluctuation in the 10–15 °C range led to a variation in the POF sensor’s response by about ±4%.
- Additional temperature increases were noted when the vehicle was in motion, likely due to the activation of other car systems like the heating system, which influenced the temperature inside the vehicle.
- Despite observing impacts of around 2–3 g, no significant signal variation in the POF sensor’s response was noted. This implies that the optical coupling between the polymer optical fiber sensors and their LED transmitter and photodiode receiver was stable and unaffected by vibrations of this magnitude.

**Figure 8:**
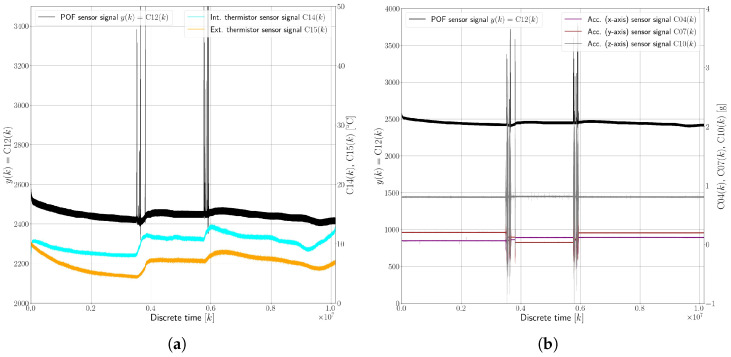
Example measurements y(k) for experiment no. 8 (F1 state) versus (**a**) temperature and (**b**) accelerations.

The dataset illustrating the oil leakage fault (F1), shown in Figure 8, spans a period of 1692 min (approximately 28 h) and provides insights into the sensor’s behavior in a scenario where 90% of the oil had already leaked out before the start of the experiment. This dataset, divided into two key visual representations, shows the correlation between the sensor’s response and the temperature (Figure 8a), and presents the sensor’s response versus measured accelerations (Figure 8b). Here, the following observations were drawn:

- There was a significant signal variation in the sensor’s response under the F1 condition, with changes of up to 50%. This is a stark contrast to the F0 state, where the sensor’s response primarily fluctuated with temperature changes.
- The most notable variations in the sensor’s response occurred during vehicle turns. This suggests that the sensor’s performance is heavily influenced by the movement and distribution of the remaining oil in the container.
- When the vehicle was inactive or in a static state, the sensor readings appeared to revert to what would be expected in the faultless state (F0). This indicates that the sensor’s performance is dependent on the oil coverage over the sensing region.
- During the vehicle’s turns, the altered distribution of the limited remaining oil caused the POF sensor to be intermittently exposed to air, as it was no longer fully submerged. This exposure led to the observed signal variations.
- The sensor’s readings returned to baseline levels when the vehicle was stationary, implying that the oil resettled over the sensor, reestablishing conditions akin to the faultless state.

**Figure 9:**
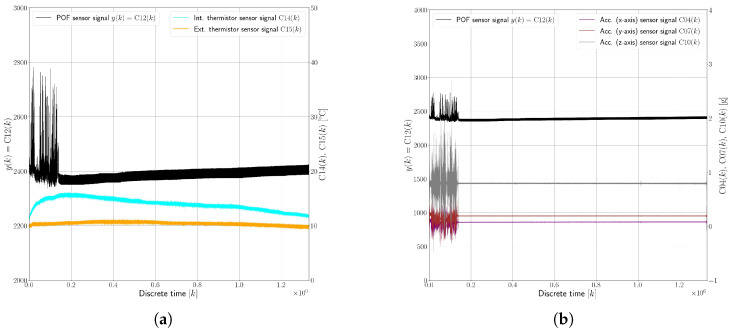
Example measurements y(k) for experiment no. 21 (F2 state) versus (**a**) temperature and (**b**) accelerations.

The dataset demonstrating the water intrusion fault (F2) is illustrated in Figure 9, focusing on the response of the sensor in relation to measured temperature (Figure 9a) and accelerations (Figure 9b). This particular dataset spans a relatively short time horizon of only 42 min, a duration chosen to provide a higher-resolution view of the sensor’s response in the presence of an oil–water emulsion, a condition resulting from the water intrusion fault, especially during the varying accelerations caused by the vehicle’s movements. Key observations from this example F2 dataset include the following:

- A noticeable signal variation of up to 20% was observed, which is less pronounced compared to the signal variation seen in the oil leakage fault scenario (F1). This difference is attributed to the distinct refractive indices the sensor encountered due to the presence of water droplets in the oil.
- The sensor registered exposure to water droplets consistently during the vehicle’s movements, irrespective of specific maneuvers like turns. This suggests that the movement of the vehicle was crucial in dispersing and revealing the presence of water droplets within the system, leading to detectable variations in the sensor’s response.
- The lack of forced circulation in the system (due to the absence of a pump, for instance) meant that the vehicle’s movement was essential for demonstrating the presence of water droplets, especially with the low fault magnitudes used in the test.
- After the vehicle came to a stop, the signal variation caused by the water droplets remained evident for a certain duration until the water settled. Once settled, the system appeared to revert to a faultless state, misleadingly indicating the absence of a fault.
- The volume of the water intrusion in these tests was not enough to continually cover the sensing area, highlighting a significant challenge in early-stage water intrusion detection. If the vehicle remains stationary and the intruded water volume is minimal, the fault might not be detected, as the water fails to consistently engage the sensor.

### 3.5. RAE-GRU Fault Detection Method

**Figure 10:**
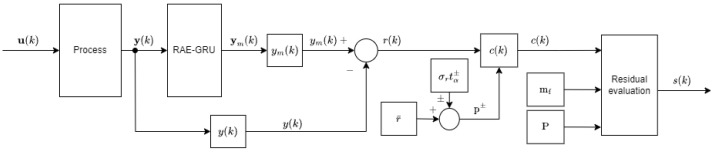
RAE-GRU model-based fault detection scheme.

The proposed fault detection method utilizes a recurrent autoencoder–gated recurrent unit (RAE-GRU) neural network (Figure 10).

The key advantages of the RAE-GRU method include reduced complexity and retention of modeling capabilities. GRU layers, as employed in the RAE-GRU network, are less complex than LSTM layers. This reduced complexity directly translates to lower computational and memory demands, making the RAE-GRU approach more feasible for implementing embedded systems with constrained resources. Despite its reduced complexity, the RAE-GRU method retains the ability to model long-term dependencies in time-series data. This is a critical feature for accurately tracking and detecting anomalies or faults over extended periods.

The proposed method begins with the processing of the output vector signal, denoted as y(k). This signal serves as the input for the RAE-GRU model:

(1) $$y\left(k\right)=\left[\begin{array}{c} y(k)\\ y(k-1)\\ \cdots \\ y(k-l) \end{array}\right],$$

where *l* denotes the number of samples prior to the most recent sample.

Upon processing the input signal sequence, the autoencoder within the RAE-GRU model generates a reconstructed output signal sequence. This is denoted as $y_{m}\left(k\right)$. The objective here is for the autoencoder to learn an efficient representation (encoding) of the input data, and then use this representation to reconstruct the output as closely as possible to the original input.

(2) $$y_{m}\left(k\right)=\left[\begin{array}{c} y_{m}\left(k\right)\\ y_{m}\left(k-1\right)\\ \cdots \\ y_{m}\left(k-l\right) \end{array}\right].$$

After reconstructing the output signal, the method proceeds by calculating the residuals, r(k). These are obtained by determining the difference between the reconstructed output signal, $y_{m}\left(k\right)$, and the original input signal, y(k). They are then compared against predetermined upper and lower thresholds, denoted as $p^{+}$ and $p^{-}$, respectively. These thresholds are established based on a statistical analysis of the residuals, incorporating the mean value of the residuals, $\bar{r}$, the standard deviation of the residuals, $\sigma_{r}$, and a selected significance level, $t_{\alpha }$, which sets the sensitivity of the fault detection process:

(3) $$p^{\pm }=\bar{r}\pm t_{\alpha }^{\pm }\sigma_{r},$$

where $\bar{r}$ and $\sigma_{r}$ are derived from the data collected under the faultless state.

To generate the threshold-crossing indicator, c(k), specific conditions are applied to the residuals. The conditions for generating c(k) can be described as follows:

(4) ck=0for   p+≥rk≥p−1otherwise.

To generate the final diagnostic signal, s(k), the method utilizes the sum of the threshold-crossing indicators, c(k), over a predefined time window, denoted as $m_{f}$. This step determines whether the observed deviations, as indicated by the threshold crossings, are sustained and significant enough to be considered a fault (by exceeding P). The process can be outlined as follows:

(5) sk=0for   P≥∑j=0mfc(k−j)1otherwise.

The diagnostic signal s(k) is the final output of the fault detection process, indicating the presence of a potential fault in the system. Although out of the scope of this research paper, the s(k) signal could be used to initiate further diagnostic procedures or take corrective actions as part of the system’s overall maintenance and safety protocols.

### 3.6. Autoencoders

Autoencoders are a special kind of feed-forward neural network designed for input reconstruction. They are typically used in unsupervised learning scenarios where the goal is to have the network output (reconstruct) an approximation of its input. A classic autoencoder comprises three main parts: the encoder, the code, and the decoder. The encoder takes the input data and compresses them into a smaller, more dense representation, known as the code. The process of encoding effectively reduces the dimensionality of the data, capturing the most salient features. The code, also referred to as the latent space representation, is the compressed version of the input data generated by the encoder. It is a lower-dimensional representation containing the input data’s key features. The decoder takes the code and attempts to reconstruct the original input data from this compressed form. The quality of the reconstruction depends on how well the encoder has captured the essential features in the code [42].

The typical training process of an autoencoder is unsupervised, meaning it does not require labeled data. The network learns to reconstruct the input data as accurately as possible to minimize the difference between the input and the output. This characteristic makes autoencoders useful for various applications such as anomaly detection, where deviations from the reconstructed normal data can signal anomalies; text generation, where they can aid in creating new text sequences; and signal denoising, where they help in removing noise from the data [43]. By utilizing a smaller number of hidden layers (or neurons) in the code compared to the number of features in the input data, the autoencoder learns a compact representation of the input. This process, known as dimensionality reduction, is essential in capturing the most relevant aspects of the data in a more efficient form [44]. To effectively handle sequential data, such as time series, text, or speech, researchers have developed recurrent autoencoders. These models adapt the basic autoencoder architecture to process sequential input. Two prominent architectures in this category are the encoder–decoder model [45] and the sequence to sequence (Seq2Seq) model [44]. Both architectures incorporate recurrent neural network (RNN) layers in the encoder and decoder components. The use of RNN layers is key in these models, as RNNs are specifically designed to process sequential data by maintaining a form of internal memory or state across the sequence.

The RAE generates an output sequence Y,

(6) $$Y=(y^{\left(0\right)},y^{\left(1\right)},\ldots ,y^{\left(n_{Y}-1\right)}),$$

for a given input sequence X:

(7) $$X=(x^{\left(0\right)},x^{\left(1\right)},\ldots ,x^{\left(n_{X}-1\right)}),$$

where $n_{X}$ is the size of the input sequence, and $n_{Y}$ is the size of the output sequence ($n_{X}$ and $n_{Y}$ can be of the same or different sizes). In many recurrent autoencoder implementations, the input sequence X is set equal to the output sequence Y; that is, X=Y. This approach is used to train the autoencoder to learn an efficient, compact representation of the input data, which is essential for effective data reconstruction.

### 3.7. Gated Recurrent Units

The gated recurrent unit, introduced by researchers in 2014 [45], is an evolution of the traditional RNN and a variant of the more complex LSTM layer. A GRU maintains the ability to effectively process sequential data while simplifying the architecture seen in LSTM layers. This simplification provides GRUs with several advantages, including fewer parameters, the absence of an output gate, quicker training times, and model simplicity. Unlike LSTM, which has three gates (input, output, and forget), a GRU combines the input and forget gates into a single “update gate” ($z_{t}$). Furthermore, it also consists of a reset gate ($r_{t}$), activation ($H_{t}$), and candidate activation (${\tilde{H}}_{t}$). $z_{t}$ is used to control how much historical information needs to be forgotten:

(8) $$z_{t}=\sigma_{g}\left(W_{z}x_{t}+R_{z}h_{t-1}+b_{z}\right).$$

The reset gate is responsible for modulating the information flow from the candidate state. It determines the extent to which the previous state (the candidate state) influences the current state:

(9) $$r_{t}=\sigma_{g}\left(W_{r}x_{t}+R_{r}h_{t-1}+b_{r}\right).$$

The candidate activation represents what can be considered as the new information at the current time step. This candidate activation is essentially a proposed update to the hidden state of the GRU, formulated based on the current input and the information retained from the previous state:

(10) $${\tilde{H}}_{t}=\tanh\left(W_{\tilde{H}}x_{t}+R_{\tilde{H}}r_{t}⊙H_{t-1}+b_{\tilde{H}}\right).$$

The activation is formed through a combination of the update gate’s output and the candidate activation. The update gate controls the balance between retaining new information and maintaining old information. Essentially, when more new information is preserved, the amount of old information factored into the current state decreases, and the opposite occurs when less new information is retained:

(11) $$H_{t}=z_{t}⊙H_{t-1}+\left(1-z_{t}\right)⊙{\tilde{H}}_{t}.$$

The matrices W, R, and b represent interconnected components crucial to the network’s functionality: input weights, recurrent weights, and biases, respectively. Each component is represented as follows:

(12) $$W=\left[\begin{array}{c} W_{z}\\ W_{r}\\ W_{\tilde{H}} \end{array}\right],R=\left[\begin{array}{c} R_{z}\\ R_{r}\\ R_{\tilde{H}} \end{array}\right],b=\left[\begin{array}{c} b_{z}\\ b_{r}\\ b_{\tilde{H}} \end{array}\right].$$

### 3.8. Model Performance Indicators

Assessing the effectiveness of process models, including those derived through deep learning, is typically conducted using a range of established methods. Commonly, ex-post measures designed for evaluating the accuracy of forecasts across various time series are utilized, regardless of their origin. These standard measures are applicable in analyzing the performance of process models generated through any modeling technique. When it comes to specifically evaluating trained deep learning models, a variety of criteria can be adopted. Drawing on the existing literature [46,47], this section details several selected criteria that are widely recognized and used for this purpose.

When evaluating the accuracy of a model, particularly in the context of deep learning, certain key metrics are frequently used, such as mean absolute error (MAE), mean absolute percentage error (MAPE), mean squared error (MSE), and root mean squared error (RMSE). The closer these values are to 0, the more accurate the model is considered to be.

However, model accuracy is not the only factor to consider. The complexity of the model also plays a crucial role in its evaluation. When developing neural models, it is important to balance accuracy with complexity. To this end, various information criteria are utilized. These criteria evaluate the trained models while taking into account their complexity. One such criterion is Akaike’s information criterion (AIC), which helps in determining the most efficient model that achieves high accuracy without unnecessary complexity.

#### 3.8.1. Mean Absolute Error (MAE)

The MAE serves as a metric for assessing the accuracy of predictive models. It measures the average size of the errors in the predictions made by the model, without considering the direction of these errors. The MAE is calculated as the average of the absolute differences between the predicted and actual values across the test dataset. In this computation, each difference between a predicted value and its corresponding true value is given equal importance, ensuring a straightforward and unbiased assessment of the model’s predictive accuracy:

(13) $$\mathrm{MAE}=\frac{1}{n_{s}N_{G}}\sum_{j=1}^{n_{s}}\sum_{i=0}^{N_{G}}\left|y_{ij}-{\hat{y}}_{ij}\right|,$$

where

- $n_{s}$ symbolizes the number of outputs in the model. It represents how many different predictions or response variables the model generates for each input;
- $y_{ij}$ is the actual value of the j-th output when the model is given the i-th input. It is the true value against which the model’s prediction is compared;
- ${\hat{y}}_{ij}$ denotes the predicted value for the j-th output based on the i-th input. It is what the model believes the output should be, based on its learning and the input it received;
- $N_{G}=\mathrm{card}\left(L_{G}\right)$ represents the total number of instances in the testing dataset. The term “cardinality” refers to the size or number of elements in the dataset. This figure is crucial for averaging the absolute errors across all predictions made by the model.

#### 3.8.2. Mean Absolute Percentage Error (MAPE)

The MAPE is a widely utilized metric for gauging the accuracy of a model’s predictions in terms of percentage. It is computed by determining the average of the absolute percentage differences between the predicted values, ${\hat{y}}_{ij}$, and the actual values, $y_{ij}$, across the test dataset. The MAPE essentially quantifies the average size of the errors in predictions as a percentage of the true values, making it a particularly useful metric in fields like forecasting and time-series analysis, where understanding the relative magnitude of errors is important:

(14) $$\mathrm{MAPE}=\frac{100\%}{n_{s}N_{G}}\sum_{j=1}^{n_{s}}\sum_{i=0}^{N_{G}}\left|\frac{y_{ij}-{\hat{y}}_{ij}}{y_{ij}}\right|.$$

#### 3.8.3. Mean Squared Error (MSE)

The MSE is a standard metric for measuring the average of the squares of errors made in a model’s predictions. Specifically, the MSE is computed as the arithmetic mean of the squared discrepancies between the model’s predicted values, ${\hat{y}}_{ij}$, and the actual true values, $y_{ij}$, within the test dataset. The use of squaring the differences in MSE means that it emphasizes larger errors more than smaller ones, providing a clear indication of the model’s precision. This property makes MSE a valuable tool for assessing the ’goodness of fit’ of a model, especially in scenarios where avoiding large errors is crucial:

(15) $$\mathrm{MSE}=\frac{1}{n_{s}N_{G}}\sum_{j=1}^{n_{s}}\sum_{i=0}^{N_{G}}{\left(y_{ij}-{\hat{y}}_{ij}\right)}^{2}.$$

#### 3.8.4. Root Mean Squared Error (RMSE)

The RMSE is a widely employed metric for assessing the performance of predictive models. It operates as a quadratic scoring rule, offering a gauge of the average size of the errors generated by a model’s predictions. The RMSE is specifically calculated by taking the square root of the MSE, which is the arithmetic mean of the squared deviations between the model’s predicted values, ${\hat{y}}_{ij}$, and the actual observed values, $y_{ij}$, in the test dataset. This calculation essentially provides a measure of the magnitude of the errors, with a particular emphasis on larger errors, due to the squaring process involved in the MSE:

(16) $$\mathrm{RMSE}=\frac{1}{n_{s}}\sum_{j=1}^{n_{s}}\sqrt{\frac{1}{N_{G}}\sum_{i=0}^{N_{G}}{\left(y_{ij}-{\hat{y}}_{ij}\right)}^{2}}.$$

#### 3.8.5. Akaike’s Information Criterion (AIC)

AIC is a widely used statistical tool for model selection, particularly when comparing the fit of different predictive models while accounting for their complexity. AIC integrates a penalty term for the number of parameters in a model, thus balancing fit quality against model complexity. A model with a lower AIC value is typically preferred, as it suggests a more efficient balance between accurately fitting the data and maintaining a simpler model structure. When evaluating multiple models, the one with the lowest AIC is generally considered the most suitable choice:

(17) $$\mathrm{AIC}=N_{G}\;\ln\left(Q_{G}\right)+2h,$$

where *h* represents the number of parameters within the model, and $Q_{G}$ is the chosen quality measure (such as RMSE) calculated for the model based on the test dataset.

#### 3.8.6. Fault Detection Performance Indicators

In evaluating fault detection performance, especially in the context of liquid intrusion and leakage, it is certainly appropriate to employ a set of indices specifically related to detection effectiveness. For this, three fundamental measures, proposed by Bartyś et al. [48], were employed:

- Detection delay time ($t_{dt}$):
  (18) $$t_{dt}=t_{dd}-t_{from},$$
  This measure quantifies the delay between the actual fault occurrence ($t_{from}$) and the time it is detected by the system ($t_{dd}$).
- False detection rate ($r_{fd}$):
  (19) $$r_{fd}=\frac{\Sigma_{n}t_{fd}^{n}}{t_{from}-t_{on}},$$
  This metric measures the ratio of time during which the system incorrectly indicates fault presence. Here, $t_{fd}^{n}$ refers to the period where the diagnostic signal wrongly suggests a system fault between $t_{on}$ and $t_{from}$.
- True detection rate ($r_{td}$):
  (20) $$r_{td}=\frac{\Sigma_{n}t_{td}^{n}}{t_{hor}-t_{from}},$$
  This formula calculates the proportion of time in which the system accurately detects faults. $t_{td}^{n}$ is the period where the diagnostic signal correctly identifies a fault within the interval from $t_{from}$ to $t_{hor}$.

## 4. Experiments and Results

### 4.1. Neural Modeling

To link the proposed fault detection method, as illustrated in Figure 10, with the designed mechatronic platform shown in Figure 4, specific relationships are established for the process input u(k), and output y(k) signals. These relationships are important to ensure that the fault detection method is properly integrated and responsive to the inputs and outputs of the mechatronic system. This involves defining how the input signals are processed and transformed into outputs by the system, and then how these outputs are fed into and analyzed by the fault detection algorithm:

(21) $$u\left(k\right)=\left[\begin{array}{c} C11(k)\\ C11(k-1)\\ \cdots \\ C11(k-l) \end{array}\right],$$

(22) $$y\left(k\right)=\left[\begin{array}{c} C12(k)\\ C12(k-1)\\ \cdots \\ C12(k-l) \end{array}\right].$$

The number of samples prior to the most recent sample *l* was chosen empirically to be four. The remaining channels, i.e., C01(k)–C03(k), C05(k), C06(k), C08(k), C09(k), and C13(k), were irrelevant for the sake of this verification study.

**Figure 11:**
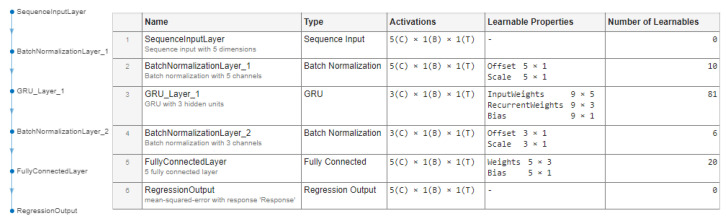
The final structure of the autoencoder neural network used for fault detection.

In the setup of the optical liquid detection system employing POF sensors, the proposed fault detection method leverages past and current POF sensor readings (denoted as C12) to learn and compress the input data into a more compact, three-dimensional representation, facilitated by the GRU layer of the network. The objective is to train the network to accurately reconstruct the original input data (represented as C11) from this compressed form. Any substantial deviations in the reconstruction error, or residuum, are potential indicators of anomalies. The architecture of this applied RAE-GRU model is shown in Figure 11.

An analysis of the signals under the F0 state indicated that variations in the signal typically occur over longer periods, with any sudden changes potentially signaling a fault. Consequently, running the fault detection algorithm at the same frequency as the data sampling rate (100 Hz) was deemed unnecessary. To address this, signal decimation was introduced, reducing the frequency of analysis. This decimation, performed at a factor of 500, effectively lowered the sample rate from 100 Hz to 0.2 Hz. This rate reduction was achieved through a low-pass Chebyshev Type I Infinite Impulse Response (IIR) filter, specifically chosen for its eighth-order and passband ripple characteristics. The selected filter efficiently preserves vital components of the signal while diminishing the impact of higher frequencies, making it an apt choice for processing the data in this context.

The artificial neural network designed for this application has a structured architecture comprising several layers, each with specific functions and parameters, as follows:

- A sequence input layer—a layer to handle sequence input data with a dimensionality of 5. It forms the network’s initial stage, receiving and processing the sequential input.
- First batch normalization layer—employing 10 learnable parameters, including an offset (5 × 1) and a scale (5 × 1), this layer normalizes the features in each batch of input data, ensuring a zero mean and unit variance.
- The first and only GRU layer—the network’s core. This layer has 81 learnable parameters. These include input weights (9 × 5), recurrent weights (9 × 3), and biases (9 × 1). It utilizes tanh and sigmoid activation functions for updating the state and gates, respectively. The layer is set to handle five input features and three hidden units in the GRU.
- The second batch normalization layer—comprising six learnable parameters, this layer is responsible for normalizing the outputs of the GRU layer, ensuring the data fed to the subsequent layer are well regulated.
- A fully connected layer—consisting of 20 learnable parameters (weights of size 5 × 3 and a bias of size 5 × 1), it is tailored for regression tasks, converting the processed data from the GRU layer into a format suitable for output.
- A regression output layer—the final layer that uses the MSE as the loss function.

During its training phase, the model employed the Adaptive Moment Estimation (ADAM) optimization method. Key parameters for this method were set as follows:

- The gradient decay factor, $\beta_{1}$, was fixed at 0.9, giving precedence to more recent gradients.
- The squared gradient decay factor, $\beta_{2}$, was set at 0.999. This value averages over the lengths of the last 1000 parameter updates.
- The epsilon value, ε, was established at $1\times 10^{-8}$ to ensure numerical stability and prevent divisions by zero in the ADAM update rule.
- The initial learning rate began at 0.01. This rate determines the step size in each iteration toward the loss function’s minimum. To adapt to the training process over time, the learning rate was reduced by a factor of 0.99 every 50 epochs.
- For handling the extensive data, the model training utilized mini-batches of $1\times 10^{11}$ samples in each iteration.
- The ‘l2norm’ method was applied to manage the gradient scale effectively.

The training of the model was conducted using two of the seven datasets recorded in F0 conditions (exps. nos. 5 and 6, as per Table 2), representing approximately 14% of the total F0 data entries. These specific datasets were chosen because they encompassed measurements from both the lower and middle ranges of the y(k) signal, and their data aligned with the median registered oil temperatures. The efficacy of the model was evaluated using dataset no. 3. To determine the final configuration of the RAE-GRU network, various architectural designs were investigated. This process involved varying the number of GRU layers (either one or two) and their corresponding hidden units. Table 3 displays the configurations along with their associated performance metrics (calculated according to Equations (13)–(17)).

In line with the principle that simpler models are less likely to overfit and generally exhibit better generalizability to new data, model ID no. 8 was chosen. This model demonstrated performance with an MAE of 1.028 and an MAPE of 0.992%, reflecting minor deviations from the actual values. The RMSE and MSE were comparatively higher, indicating that larger errors were more heavily penalized. The AIC scores suggested a well-balanced model in terms of complexity. For instance, while model no. 3 showed superior performance based on the MAE and RMSE metrics, its AIC was over five times greater, indicating a considerably higher complexity. Model no. 1 emerged as a viable alternative to model no. 3, primarily due to its reduced complexity (AIC of 204). Nevertheless, model no. 3 was ultimately selected owing to its lower MAPE, which did not surpass 1.0%.

**Figure 12:**
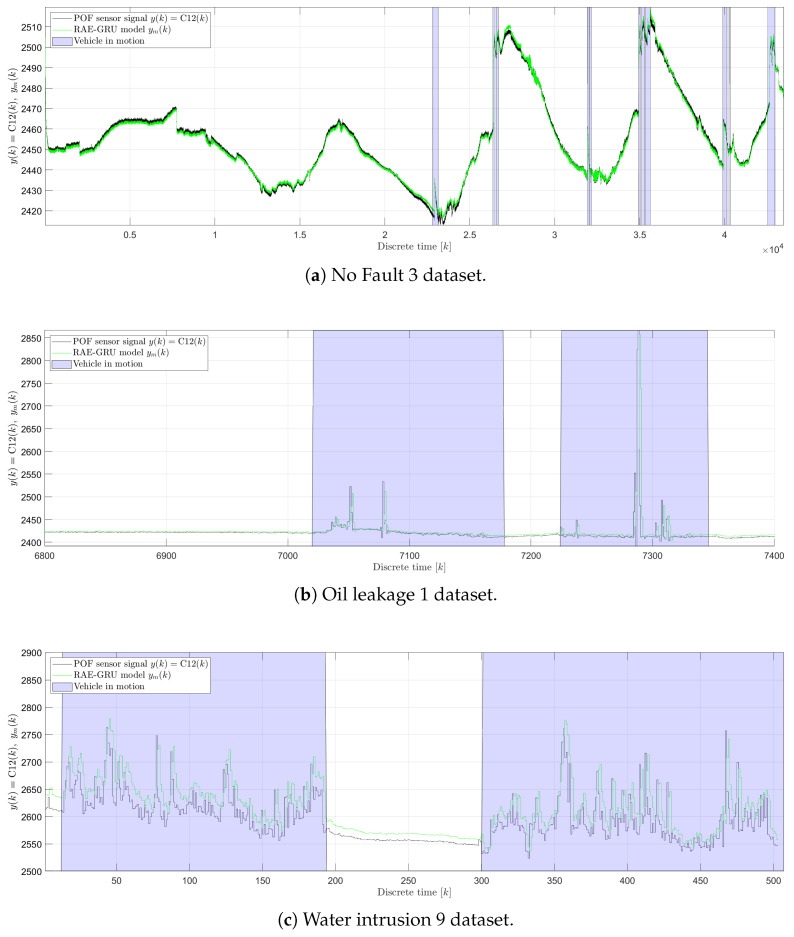
Example POF sensor and REA-GRU model output signals.

Figure 12 presents sample outputs from the POF sensor alongside the corresponding outputs from the RAE-GRU model for various states. This illustration includes testing and verification data as detailed in Table 2. Specifically, it shows testing data from experiment no. 4 for the faultless state (Figure 12a), along with verification data for the liquid leakage and liquid intrusion states. Data for the liquid leakage state from experiment no. 8 (F1 state) are shown in Figure 12b, and data for the liquid intrusion state from experiment no. 21 (F2 state) are shown in Figure 12c. In addition, periods when the vehicle was in motion are highlighted in blue, indicating active vehicle states. These motion states were determined post hoc from acceleration data captured by sensors C04(k), C07(k), and C10(k). The output signals from the RAE-GRU model, denoted as $y_{m}\left(k\right)$, are designed to mirror the readings from the polymer optical fiber sensor, denoted as y(k)=C12(k). However, discrepancies such as signal offsets are noticeable, particularly in scenarios involving water intrusion, as illustrated in Figure 12c. Furthermore, under conditions of liquid leakage and water intrusion, fluctuations occurring during vehicle movement due to the presence of faults are also captured by the RAE-GRU model, albeit with diminished amplitude. These features underscore the model’s capability to reconstruct and interpret dynamic changes in sensor data during different operational states.

**Figure 13:**
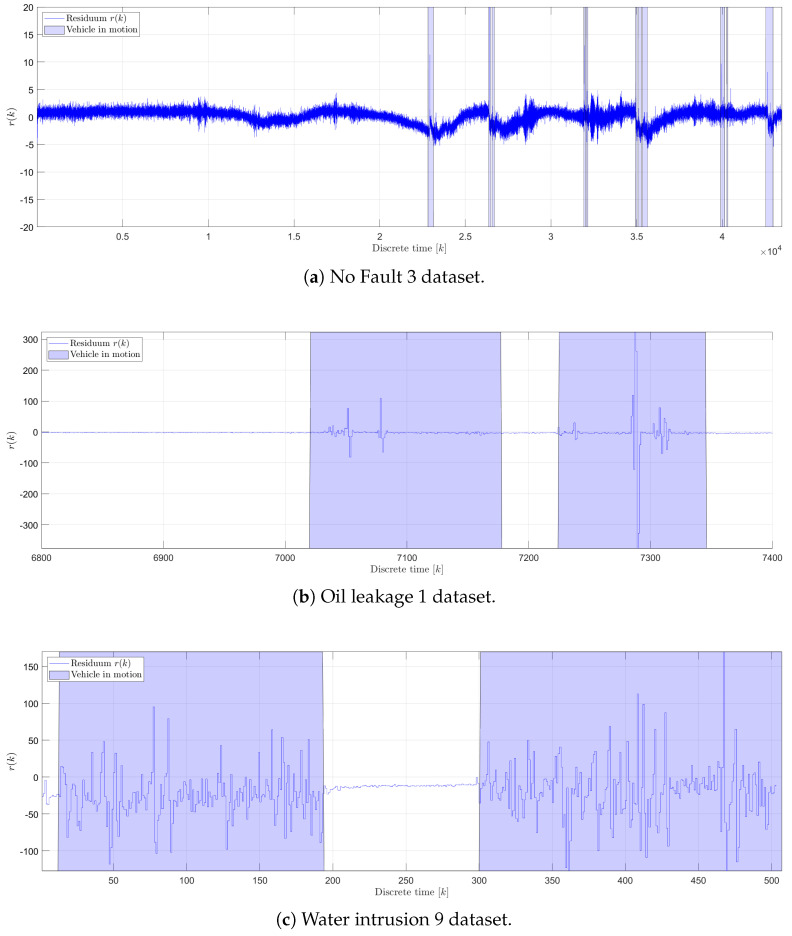
Example generated residuum signals.

Figure 13 illustrates the residuum r(k) generated by the RAE-GRU model, showcasing data across different operational states: the faultless state (Figure 13a), the oil leakage fault (Figure 13b), and the water intrusion fault (Figure 13c). In the faultless state, there is a slight correlation between the vehicle’s activity and the residuum, indicating minor fluctuations that correspond with the vehicle’s motion.

For both fault conditions, the residuum tends to be close to zero when the vehicle is stationary. However, as the vehicle begins to move, fluctuations in the residuum are observed, which are indicative of the system’s response to operational anomalies. The amplitude of these fluctuations differs significantly depending on the type of fluid exposure the POF sensor encounters. Specifically, during the oil leakage fault, the sensor’s exposure to air results in r(k) amplitudes that are up to ten times higher than those observed during the water intrusion fault, where the sensor encounters a water–oil emulsion. This highlights the sensitivity of the sensor to different environmental conditions and the capability of the RAE-GRU model to detect and respond to these variations in real time. The employed fault detection method relies on two key statistical measures, $\bar{r}$ (the mean of the residuum) and $\sigma_{r}$ (the standard deviation of the residuum), along with three predefined parameters: $t_{\alpha }$, $m_{f}$, and $t_{s}$. The parameters $\bar{r}$ and $\sigma_{r}$ are used to set the upper ($p^{+}$) and lower ($p^{-}$) detection thresholds and were derived from the statistical analysis of the residuum data from the testing dataset. Specifically, $\bar{r}$ was determined to be 0.133478 and $\sigma_{r}$ was 1.425754. The significance level $t_{\alpha }$, which influences the breadth of the threshold band, was empirically set at 24. This setting helps determine the sensitivity of the fault detection, influencing how outlier-driven the model’s response should be. Additionally, the parameters related to the generation of the diagnostic signal, $m_{f}$ (the memory factor) and P (the threshold crossing count), were configured to 3 and 5, respectively. This configuration ensures that a final diagnostic signal will be issued if the generated r(k) crosses the defined thresholds at least five times within a span of two minutes. This time frame is derived by setting P equal to 24, resulting in 120 s, given that the time interval between residual data samples post-decimation is 5 s. This setup ensures a balance between responsiveness and reliability, mitigating false positives while ensuring timely fault detection.

### 4.2. Overall Results of Fault Detection

**Figure 14:**
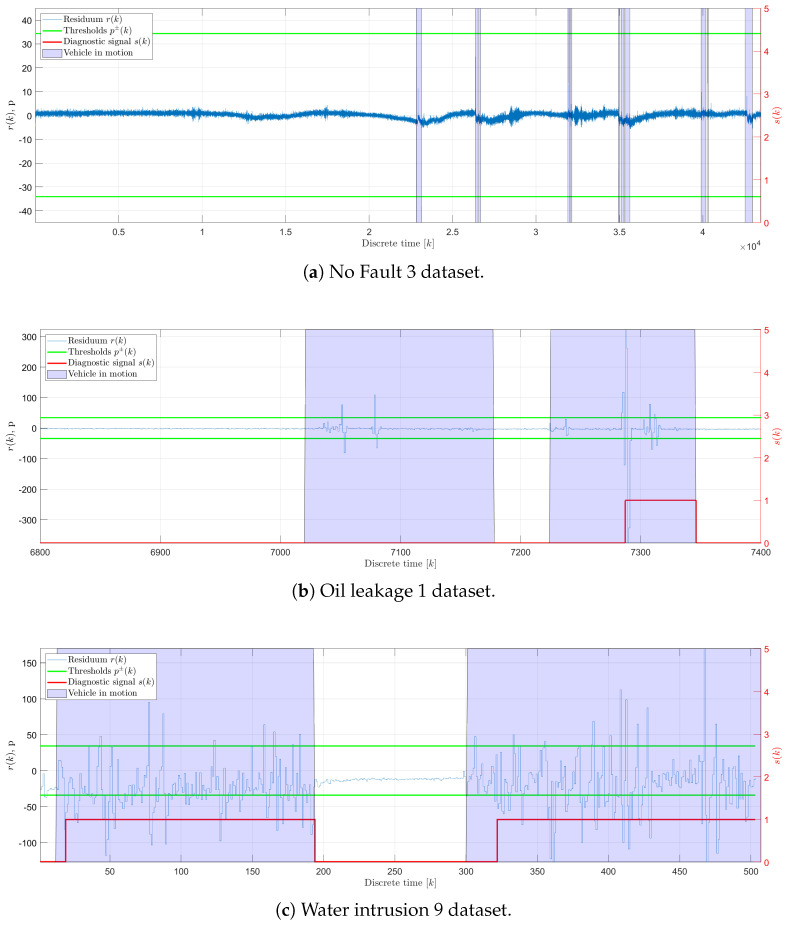
Example results of the fault detection method.

Figure 14 presents the results of the fault detection method using autoencoder neural networks (Figure 10). The results illustrate that the upper and lower thresholds were accurately set, given that the generated residuals remain within these limits in a faultless state.

It is also evident that irrespective of the vehicle’s motion state, the magnitude of the fault is not significant enough to produce noticeable variations in the polymer optical fiber sensor’s signal y(k). However, once the vehicle initiates motion, fluctuations in both the observed signal y(k) and its corresponding residuum r(k) are apparent, leading the fault detection method to identify faults in both signals. Therefore, faults are most likely to be detected when the vehicle is in motion. Because of that, traditional performance indices such as $r_{td}$ and $t_{dt}$ (as defined in Equations (19) and (20)) proved to be unreliable for this method. Consequently, new indices, $r^{\prime }td$ and $t^{\prime }dt$, were derived to better evaluate the method’s performance. These new indices are calculated based on periods of vehicle motion, as indicated by accelerometer data that accompany each dataset. The detection rates and delay times are computed for each period of motion and then averaged (${\bar{r}}^{\prime }td$ and ${\bar{t}}^{\prime }dt$) for the dataset. The summarized results of these evaluations, including false and true detection rates as well as detection delay times for each motion state, are detailed in Table 4. This table provides a comprehensive overview of the method’s performance across different operational scenarios, highlighting its effectiveness and reliability under dynamic conditions.

The results revealed that the fault detection method that uses the RAE-GRU model reported no false positives, as indicated by a consistent ${\bar{r}}_{fd}$ of zero across all evaluated datasets. This absence of erroneous fault detection is particularly advantageous within the automotive sector, where the occurrence of false positives can lead to unwarranted maintenance actions and impose unnecessary burdens on vehicle operators. On the other hand, the detection delay time, ${\bar{t}}_{dt}$, is irrelevant under F0 conditions due to the lack of any faults to detect. This underscores the efficiency of the method in differentiating between normal operational states and potential anomalies. The adaptation of indices such as ${\bar{r}}^{\prime }td$ and ${\bar{t}}^{\prime }dt$ improves the evaluation of the performance of the system, providing a more granular insight into its functionality under various operational conditions. This ensures a comprehensive understanding of the system’s effectiveness, particularly in dynamically challenging environments, where traditional metrics might fail to capture the nuances of system behavior accurately.

## 5. Discussion

The fault detection method proved to be capable of identifying faults effectively when the vehicle was in motion, achieving an average true detection rate (${\bar{r}}^{\prime }td$) of 77.1%. The highest performance was recorded during experiment no. 2, with an ${\bar{r}}^{\prime }td$ of 97.2%, while the lowest was in experiment no. 1, at 60.9%. Regarding detection delay times, the method demonstrated an average ${\bar{t}}^{\prime }dt$ of 108.2 s. The shortest detection time was 25 s, in experiment no. 9, while the longest was 170 s, in experiment no. 11. These results indicate that the average detection delay ${\bar{t}}^{\prime }dt$ comfortably met the initially assumed operational requirement of 300 s. Furthermore, in experiments no. 9 and 11, the true detection rate exceeded the requisite 80% threshold, highlighting the efficacy of the fault detection approach under these conditions. The 300 s detection delay was selected as it is the only existing regulatory requirement, independent of fault severity. Currently, no additional standards specify or recommend different detection delay times for battery packs. Future research should explore these delays further and refine detection mechanisms to differentiate minor faults from critical failures, enhancing overall safety measures.

In contrast, the detection performance under the water intrusion fault state exhibited significant variability across different experiments. Initial experiments involving a fault magnitude of 0.3% concentration (50 mL of intruded water) failed to trigger the diagnostic signal s(k), indicating challenges in detecting very subtle faults. Subsequent experiments with a 0.9% concentration (150 mL of intruded water) showed that the fault was detected only towards the end of the experiment (after approximately 52 min), resulting in an ${\bar{r}}^{\prime }td$ of barely 9%. The detection metrics improved substantially when the water concentration was doubled to 1.8% (300 mL of intruded water), where the fault detection method averaged an ${\bar{r}}^{\prime }td$ of 89%. This performance met the assumed non-functional requirements of an 80% detection rate and a 300 s detection time in four out of five experiments at this fault magnitude. However, experiment no. 17 slightly fell short, with an ${\bar{r}}^{\prime }td$ of 79.6%. The highest ${\bar{r}}^{\prime }td$ achieved was 96.9%, in experiment no. 18. Notably, the average detection delay for the water intrusion state with a 1.8% fault magnitude was 98 s, which was approximately 10% quicker than that observed in the oil leakage state, likely due to the different dynamics involved in each fault type.

The indices $r^{\prime }td$ and $t^{\prime }dt$ were calculated for each motion state experienced during the experiments. Table 5 and Table 6 present the detection delay time and true detection rates, respectively, for each vehicle-in-motion state during the experiments. These tables underscore the variable nature of each test, reflecting the realistic and unpredictable driving conditions, and the lack of driving patterns during the performed experiments.

Further analysis of the data under the oil leakage fault revealed that the fault detection method effectively recognized the presence of a fault in 21 out of 22 instances when the vehicle was in motion. The detection delay for these instances exhibited a significant range, from as short as 20 s to as long as 640 s. The true detection rate ($r_{td}^{\prime }$) for these detections varied substantially, ranging from 33.8% to 97.2%, reflecting variability in the method’s responsiveness and accuracy under different conditions.

In scenarios involving the water intrusion fault with a weight-to-weight concentration of 1.8%, the detection method consistently identified the fault every time the vehicle was in motion. The detection delay for this fault type was notably shorter, ranging from a minimum of 5 s—a constraint set by the data decimation process—to a maximum of 185 s. The true detection rate for these conditions spanned from 79.6% to 96.0%, indicating a generally high level of performance.

This paper lays a strong foundation for advancing the technology readiness level of the optical liquid detection system used within electric vehicle battery packs. Several critical areas have been identified for future research to enhance the system’s robustness, integration capabilities, and overall performance in diverse operational environments. These are essential for transitioning from a fully functional prototype verified under real conditions to a fully integrated system ready for widespread application in the automotive industry.

- The initial evaluation of the developed fault detection methods was conducted offline, using real-world datasets collected under road conditions. To further improve the robustness and reliability of the system, future studies should explore a wider array of test environments. This includes testing under more extreme weather conditions and varied driving scenarios, which may affect the system’s performance. For instance, testing in colder climates, where low temperatures could affect the viscosity and behavior of the cooling liquid, or in hotter climates, where overheating could lead to different fault signatures. Such comprehensive testing will help ensure that the system can perform reliably under all possible operating conditions encountered in global automotive markets.
- Integrating the OLDS with other critical components of the battery pack, such as the BMS and the BTM system, represents a logical progression in the system’s development. This integration would allow for holistic management of the battery pack’s health and operational status, enhancing fault detection capabilities during periods of vehicle inactivity by maintaining forced liquid flow. Further, integrating these systems addresses challenges associated with computational constraints and real-time processing demands. Subsequent verification studies using this integrated approach would provide valuable insights into the system’s efficacy and inform adjustments needed to optimize performance.
- The interdisciplinary nature of the challenges faced by the OLDS suggests that future research could benefit significantly from a cross-disciplinary approach. Combining insights from mechanical engineering, optics, materials science, electrical engineering, computer science, and artificial intelligence could lead to substantial improvements in system design and functionality. For instance, material scientists could develop more efficient and resilient sensor materials, while computer scientists could enhance algorithms for faster and more accurate data processing. Within the realm of artificial intelligence, exploring alternative AI models could optimize the system’s ability to process data and make real-time decisions. Investigating the application of emerging AI techniques like deep learning or reinforcement learning could provide the OLDS with enhanced predictive capabilities, adapting more effectively to the complex dynamics of battery operation and fault evolution.

## 6. Conclusions

The findings confirm that the developed polymer optical fiber sensor is effective for detecting both water intrusion and oil leakage faults. The extensive testing conducted has validated the robustness of the designed and prototyped optical liquid detection system for the purposes outlined in this paper. Given the relatively low values of the generated residuum r(k)—amounting to approximately 1–2% of the POF sensor’s observed signal y(k) in the faultless state—it can be concluded that the RAE-GRU model of the proposed method has adeptly learned the long-term dependencies experienced under road conditions. The residuum evaluation approach implemented has resulted in zero false detection alarms across all test datasets, underscoring the method’s reliability.

The efficacy of the proposed fault detection method is notably influenced by the magnitude of the fault, illustrating a direct correlation between fault magnitude and detection performance. Additionally, the absence of mechanisms to facilitate forced liquid flow within the system means that the efficacy of the fault detection is tied to the vehicle’s movement. This dynamic is due to the placement of the sensor at the base of the container, mounted on small ribs, which could potentially limit the system’s sensitivity under certain conditions. Specifically, in cases of water intrusion, the fault magnitude must be significant enough for the water to reach the sensor under static conditions. If the volume of intruding water is too low, it may not make contact with the sensor unless the vehicle is in motion, causing the water droplets from the oil–water emulsion to circulate and eventually reach the detection threshold. This scenario highlights a critical area for potential enhancements in sensor placement or the introduction of active circulation mechanisms to improve fault detection sensitivity under a wider range of operational conditions.

## 7. Patents

The optical liquid detection system described in this document has been the subject of a patent application, published under the number WO2023169875. The patent, titled “OPTICAL LIQUID DETECTION SYSTEM”, is currently in the application stage.

## Figures and Tables

**Table 1:** Considered technical states.

State ID	State Description
F0	Faultless state
F1	Oil leakage
F2	Water intrusion

**Table 2:** Experiment scenarios.

Exp. ID	Data Type	State ID	Description	Mag. [%]	Q_1_ [mL]	t_1_ [min]	t_2_ [min]
1	Verification	F0	No fault 0	-	-	2596	104
2	Verification	F0	No fault 1	-	-	1873	127
3	Verification	F0	No fault 2	-	-	3468	274
4	Testing	F0	No fault 3	-	-	3623	176
5	Training	F0	No fault 4	-	-	1061	70
6	Training	F0	No fault 5	-	-	907	51
7	Verification	F0	No fault 6	-	-	562	-
8	Verification	F1	Oil leakage 1	90	15,000	1692	51
9	Verification	F1	Oil leakage 2	90	15,000	211	15
10	Verification	F1	Oil leakage 3	90	15,000	726	27
11	Verification	F1	Oil leakage 4	90	15,000	1932	89
12	Verification	F1	Oil leakage 5	90	15,000	1299	11
13	Verification	F2	Water intrusion 1	0.3	50	684	65
14	Verification	F2	Water intrusion 2	0.3	50	860	10
15	Verification	F2	Water intrusion 3	0.9	150	344	24
16	Verification	F2	Water intrusion 4	0.9	150	1098	91
17	Verification	F2	Water intrusion 5	1.8	300	122	10
18	Verification	F2	Water intrusion 6	1.8	300	31	27
19	Verification	F2	Water intrusion 7	1.8	300	62	26
20	Verification	F2	Water intrusion 8	1.8	300	221	21
21	Verification	F2	Water intrusion 9	1.8	300	42	32

**Table 3:** Model performance indicators for various GRU structures.

Model ID	GRU_Layer_1	GRU_Layer_2	MAE	MAPE	MSE	RMSE	AIC
1	1	3	1.91	1.84	5.83	2.41	204.00
2	5	3	1.88	1.81	5.26	2.29	564.00
3	10	3	0.83	0.80	1.32	1.15	1284.00
4	1	5	0.68	0.66	0.80	0.90	352.00
5	3	5	2.78	2.68	9.21	3.03	532.005
6	5	5	1.84	1.77	3.94	1.99	760.00
7	10	5	0.59	0.57	0.67	0.82	1460.00
**8**	**3**	-	**1.03**	**0.99**	**1.70**	**1.30**	**234.00**
9	5	-	1.46	1.41	3.39	1.84	430.00
...							
10	10	-	2.24	2.16	5.63	2.37	1130.00

**Table 4:** Performance measures for fault detection method corresponding to Figure 10.

Exp. ID	State ID	Dataset Description	${\bar{r}}_{fd}$	${\bar{r}}_{td}^{\prime }$	${\bar{t}}_{dt}^{\prime }$ [s]	Mag. [%]
1	F0	No fault 0	0.000	-	-	-
2	F0	No fault 1	0.000	-	-	-
3	F0	No fault 2	0.000	-	-	-
4	F0	No fault 3	0.000	-	-	-
5	F0	No fault 4	-	-	-	-
6	F0	No fault 5	-	-	-	-
7	F0	No fault 6	0.000	-	-	-
8	F1	Oil leakage 1	0.000	0.609	133	90
9	F1	Oil leakage 2	0.000	0.972	25	90
10	F1	Oil leakage 3	0.000	0.797	160	90
11	F1	Oil leakage 4	0.000	0.800	170	90
12	F1	Oil leakage 5	0.000	0.678	53	90
13	F2	Water intrusion 1	0.000	0.000	-	0.3
14	F2	Water intrusion 2	0.000	0.000	-	0.3
15	F2	Water intrusion 3	0.000	0.000	-	0.9
16	F2	Water intrusion 4	0.000	0.086	2100	0.9
17	F2	Water intrusion 5	0.000	0.796	48	1.8
18	F2	Water intrusion 6	0.000	0.969	50	1.8
19	F2	Water intrusion 7	0.000	0.882	185	1.8
20	F2	Water intrusion 8	0.000	0.889	140	1.8
21	F2	Water intrusion 9	0.000	0.931	68	1.8

**Table 5:** Detection delay time $t_{dt}^{\prime }$ under vehicle motion.

	$t_{dt}^{\prime }$ for Subsequent Vehicle-in-Motion States [s]									
Dataset										
description	1	2	3	4	5	6	7	8	9	10
Oil leakage 1	-	310	30	85	105					
Oil leakage 2	25									
Oil leakage 3	160	160								
Oil leakage 4	55	50	70	225	85	20	225	105	640	225
Oil leakage 5	75	50	35	50						
Water intrusion 1	-	-	-							
Water intrusion 2	-	-								
Water intrusion 3	-	-	-							
Water intrusion 4	-	-	2100							
Water intrusion 5	70	5	70							
Water intrusion 6	50									
Water intrusion 7	185									
Water intrusion 8	140									
Water intrusion 9	30	105								

**Table 6:** True detection rate $r_{td}^{\prime }$ under vehicle motion.

	$r_{td}^{\prime }$ for Subsequent Vehicle-in-Motion States									
Dataset										
description	1	2	3	4	5	6	7	8	9	10
Oil leakage 1	0.000	0.527	0.760	0.760	0.883	0.876				
Oil leakage 2	0.972									
Oil leakage 3	0.759	0.834								
Oil leakage 4	0.917	0.882	0.932	0.793	0.653	0.840	0.918	0.840	0.883	0.338
Oil leakage 5	0.595	0.600	0.720	0.796						
Water intrusion 1	0.000	0.000	0.000							
Water intrusion 2	0.000	0.000								
Water intrusion 3	0.000	0.000	0.000							
Water intrusion 4	0.000	0.000	0.257							
Water intrusion 5	0.714	0.960	0.714							
Water intrusion 6	0.969									
Water intrusion 7	0.882									
Water intrusion 8	0.889									
Water intrusion 9	0.967	0.896								

## Data Availability

The data presented in this study were newly generated but cannot be shared due to confidentiality agreements with the company.

## Abbreviations

The following abbreviations are used in this manuscript:

AIC	Akaike’s information criterion
BEV	Battery electric vehicle
BMS	Battery management system
BTM	Battery thermal management
ECU	Electronic control unit
EV	Electric vehicle
GRU	Gated recurrent unit
GTR	Global Technical Regulation
IIR	Infinite Impulse Response
LIB	Lithium-ion battery
LSTM	Long short-term memory
MAE	Mean average error
MAPE	Mean average percentage error
ML	Machine learning
MSE	Mean squared error
OLDS	Optical liquid detection system
POF	Polymer optical fiber
RAE	Recurrent autoencoder
RMSE	Root mean squared error
RNN	Recurrent neural network
SOC	State of charge
SOH	State of health
